# Outcomes of 23-Gauge Transconjunctival Sutureless Vitrectomy in Patients with Diabetic Retinopathy

**DOI:** 10.14744/bej.2021.38278

**Published:** 2021-09-27

**Authors:** Isil Pasaoglu, Mehmet Cakir

**Affiliations:** 1. University of Health Sciences, Beyoglu Eye Training and Research Hospital, Istanbul, Turkey; 2. Turkiye Hospital, Istanbul, Turkey

**Keywords:** 23-gauge pars plana vitrectomy, diabetic retinopathy, sutureless vitrectomy

## Abstract

**Objectives:**

This study evaluated the anatomical and functional results of 23-G transconjunctival sutureless vitrectomy (TSV) in diabetic retinopathy (DR) patients with a variety of vitreoretinal diseases.

**Methods:**

Consecutive patients who underwent 23-G TSV for complications of DR were evaluated retrospectively. The primary outcome measures were best-corrected visual acuity (BCVA), intraocular pressure (IOP), and intraoperative and postoperative complications.

**Results:**

A total of 42 eyes of 41 patients were included and followed up for a mean of 15.64±10.0 months. The mean patient age was 59.33±7.4 years. Indications for surgery were nonclearing vitreous hemorrhage (VH) (n=10), tractional retinal detachment (TRD) (n=8), TRD+VH (n=12), epiretinal membrane (n=5), diabetic macular edema (n=3), submacular hemorrhage (n=2), macular hole (n=1), or vitreomacular traction (n=1). There was a significant improvement in the BCVA at the postoperative first and third months, and at the last visit compared with the preoperative value (p<0.001). There was no significant change in the mean IOP measured on the postoperative first day, first week, first month, third month, or the last visit (p>0.05). In the postoperative period, the VH resolved spontaneously in 9 eyes. Repeat 23-G vitrectomy was performed in 6 eyes: 4 with recurrent retinal detachment and 2 with VH.

**Conclusion:**

The results indicate that 23-G TSV is an effective technique for vitreoretinal disease in patients with DR.

## Introduction

Diabetic retinopathy (DR) is one of the common causes of blindness in population between the ages 20 and 65 years in the world (1). Many risk factors have been investigated in the development of DR and progression to proliferative form. As a result of long-term follow-up, high glycosylated hemoglobin level, hypertension, hyperlipidemia, and nephropathy have been shown to be important and determinant risk factors (2). Retinopathy develops in almost all insulin-dependent type 1 diabetes patients and in more than 60% of insulin-independent type 2 diabetes patients within 20 years of diagnosis (3).

In proliferative diabetic retinopathy, which is characterized by neovascularization and fibrous tissue formation, pars plana vitrectomy (PPV) is performed to remove ocular media opacities, eliminate vitreoretinal tractions, and change the course of the proliferative process by removing the posterior hyaloid membrane (4). Machemer et al. performed PPV for the 1st time in the 1970s to eliminate persistent vitreous hemorrhage (VH) (5). Through the technical developments and instrumentation, indications for vitrectomy have expanded and the surgical instruments have been made smaller and functional. A 25-gauge (G) transconjunctival sutureless vitrectomy (TSV) system which has been developed by Fujii et al. has been reported to reduce surgically induced trauma, shorten the duration of surgery, and speed up the recovery in the post-operative period (6). After the 25G TSV system, the flexibility of the surgical instruments has been reduced and the 23G TSV system has been developed which allows more ocular rotation (7). The aim of this study was to evaluate the anatomic and functional results of 23G TSV technique applied in the treatment of vitreoretinal diseases due to DR.

## Methods

A retrospective chart review was performed and 41 consecutive patients (42 eyes) who underwent 23G TSV for the complications of DR between March 2006 and March 2010 in the retina department of a tertiary eye hospital were enrolled. The study was conducted in accordance with the Declaration of Helsinki principles, and the local medical ethics committee approved the research (E-48670771-514.10). Written and informed consent was obtained from all participants. Eyes with previous vitrectomy surgery and the patients who were followed less than 3 months were excluded from the study.

All patients underwent comprehensive ophthalmologic examination including best-corrected visual acuity (BCVA) using Snellen charts, slit-lamp biomicroscopy, intraocular pressure (IOP) measurement using Goldmann applanation tonometry, and biomicroscopic fundoscopy with a 90-D lens. Optical coherence tomography (OCT) was performed to visualize the macula. In cases where fundus imaging was not possible, and for the eyes with VH, B-scan ultrasonography was performed.

### Surgical Technique

All operations were performed under subtenon or general anesthesia. Before beginning PPV, phacoemulsification and intraocular lens implantation through clear corneal incision were performed in eyes with cataracts. All patients underwent 23G TSV with 360° of vitreous base shaving under scleral depression using a non-contact wide-angle viewing system (EIBOS). In all eyes, the posterior hyaloid was checked with intravitreal triamcinolone, and the adherents were removed from the retinal surface. In the eyes, where vitrectomy was performed due to epiretinal membrane (ERM), macular hole (MH), diabetic macular edema (DME) with tight posterior hyaloid, and vitreomacular traction (VMT), the membrane and the internal limiting membrane (ILM) were made visible with diluted triamcinolone acetonide and peeled off from the surface of the retina with 23G microforceps. All fibrous membranes were removed in eyes with tractional retinal detachment (TRD). Bimanual surgery was performed in three eyes using chandelier light. In all cases, lasers were completed in a scatter fashion at the end of the surgery. Air, sulfur hexafluoride (SF6, 20%), perfluoropropane (C3F8, 14%), or silicone oil were used as tamponade. At the end of the operation, after the microcannula was removed, the intravitreal tamponade leakage and the ocular tonus were checked. Sclerotomy sites were sutured if needed. Subconjunctival antibiotic and steroid injections were performed.

Study parameters included demographic data, indications for surgery, BCVA, IOP, lens status, intraoperative and post-operative complications, and follow-up time. BCVA and IOP were documented preoperatively and on post-operative 1st day, 1st week, 1st month, 3rd month, and the last visit. Hypotony was defined as IOP less than 10 mmHg, and severe hypotony as IOP less than 5 mmHg. Transient hypotony was defined as hypotonia of lasting less than 1 week.

Snellen visual acuity was converted into logarithm of the minimum angle of resolution (logMAR) for statistical analysis. Descriptive statistics were performed including means and standard deviations for samples of normally distributed variables. Repeated measure ANOVA was used for statistical assessment of IOP measurements. The paired sample t-test was used for continuous parametric data, and p<0.05 was considered to be statistically significant.

## Results

A total of 42 eyes of 41 patients were included in the study, and 20 (48.78%) patients were female. The mean age of the patients was 59.33±7.4 (range, 47–76 years) during surgery. The patients were followed for a mean of 15.64±10.0 months. Indications for surgery included non-clearing VH (n=10), TRD (n = 8), TRD + VH (n=12), ERM (n=5), DME n=3), submacular hemorrhage (SMH) (n=2), MH (n=1), and VMT (n=1).

Tamponade with SF6 was used in seven eyes (one VH, two TRD, three TRD+VH, one SMH), C3F8 in two eyes (two TRD+VH), silicone oil in 13 eyes (one VH, five TRD, five TRD+VH, one SMH, and one MH), and air in 12 eyes (five VH, two TRD+VH, two ERM, and three DME). In cases for which long-term tamponade was not needed, a partial or minimal air exchange was used in eight eyes.

PPV was successfully completed in all patients. Of the total cases, a single 23G sclerotomy site showing leakage in eight eyes and three sclerotomy sites showing leakage in three eyes were transconjunctivally sutured with 8/0 Vicryl at the end of the surgery.

It was not needed to switch over to the 20G standard vitrectomy during the operation in any case.

Thirty-one eyes were phakic, 11 eyes were pseudophakic in the study group. Before starting vitrectomy, cataract extraction was performed on seven eyes whose fundus was not adequately visualized due to lens opacity, and an intraocular lens was placed in the bag. In the post-operative follow-up time, worsening of cataract was occurred in 23 eyes and 15 of these eyes needed cataract surgery and intraocular lens implantation.

While the mean overall BCVA (logMAR) was 2.07±0.99 preoperatively, it was 2.37±0.88 at the post-operative 1st day, 1.30±0.92 at 1st month, 1.37±0.95 at 3^rd^ month, and 1.10±0.97 at the last visit. There was a significant improvement in BCVA values obtained on post-operative 1st and 3rd months and at the last visit compared to the pre-operative values (p<0.001). Pre-operative and post-operative BCVA values of the eyes in different surgical indications are summarized in Table 1.

**Table 1.** Preoperative and postoperative BCVA values in different surgical indications

Indications for Surgery	n (%)	Preoperative BCVA (logMAR)	Postoperative BCVA at last follow-up (logMAR)	p
Vitreous Hemorrage	10 (23.8)	2.67±0.71	0.69±0.36	<0.001*
Tractional retinal detachment	8 (19.0)	1.68±0.89	1.63±1.17	0.09
Tractional retinal detachment with Vitreous Hemorrage	12 (28.6)	2.76±0.54	1.69±1.22	0.01*
Epiretinal membrane	5 (11.9)	0.56±0.29	0.34±0.23	0.07
Diabetic macular edema	3 (7.1)	1.36±	0.70±0.17	0.01*
Submacular hemorrhage	2 (4.8)	1.60±1.14	2.25±1.06	‡
Vitreomacular traction	1 (2.4)	0.7	0.5	‡
Macular hole	1 (2.4)	3.00	3.00	‡

n: number of eyes; BCVA: best corrected visual acuity; logMAR: logarithm of the minimum angle of resolution; * Indicates statistically significant value; ‡Insufficient sample size for statistical analysis.

Compared to the mean pre-operative value, there was no significant change in the mean IOP in the post-operative 1^st^ day, 1^st^ week, 3^rd^ month, and last visit in the whole study group (p>0.05) (Table 2). In the post-operative follow-up time, an increase in IOP was observed in 11 eyes, which could be controlled by medical antiglaucoma treatment. Transient hypotonia (IOP <10 mmHg) occurred in four eyes and transient severe hypotonia (IOP <5 mmHg) in one eye on the 1st post-operative day. However, none of them had permanent hypotonia and IOP spontaneously increased in all eyes within 1 week. No related complications such as choroidal detachment or maculopathy were observed.

**Table 2.** Changes of IOP at Postoperative Follow-up Periods

	Intraocular pressure (mmHg)	p
Preoperative	14.78±2.78	-
Postoperative		
1 day	15.61±7.17	0.443
1 week	14.95±4.47	0.787
1 month	16.26±3.01	0.006*
3 month	16.07±4.88	0.120
Last visit	15.90±3.68	0.138

Values are presented as mean±SD; IOP, intraocular pressure; * Indicates statistically significant value.

In the early post-operative period, VH developed in nine eyes, and all were resolved spontaneously within 1 month.

In 10 eyes which were operated for VH, the mean post-operative visual acuity improved significantly compared to the pre-operative value (p<0.001) (Table 1). One eye developed recurrent VH occurred at the post-operative 6^th^ month and revitrectomy was performed with 23G TSV. At the last visit, the retina was flat with the silicone oil tamponade.

In eight eyes with TRD, there was no significant improvement in post-operative visual acuity (p>0.05) (Table 1). In two eyes, recurrent retinal detachment developed at the post-operative 1^st^ month and revitrectomy with silicone oil tamponade was performed. Retina was attached and flat in both eyes at the last visit. In two eyes, serous macular detachment was observed to continue in OCT, but reoperation was not recommended.

Three out of 12 eyes which were operated for TRD+VH developed recurrent retinal detachment at the post-operative 3^rd^ month, 23G revitrectomy with silicone oil tamponade was performed in two of these three eyes. Retina was attached and flat at the last visit.

**Figure 1.**
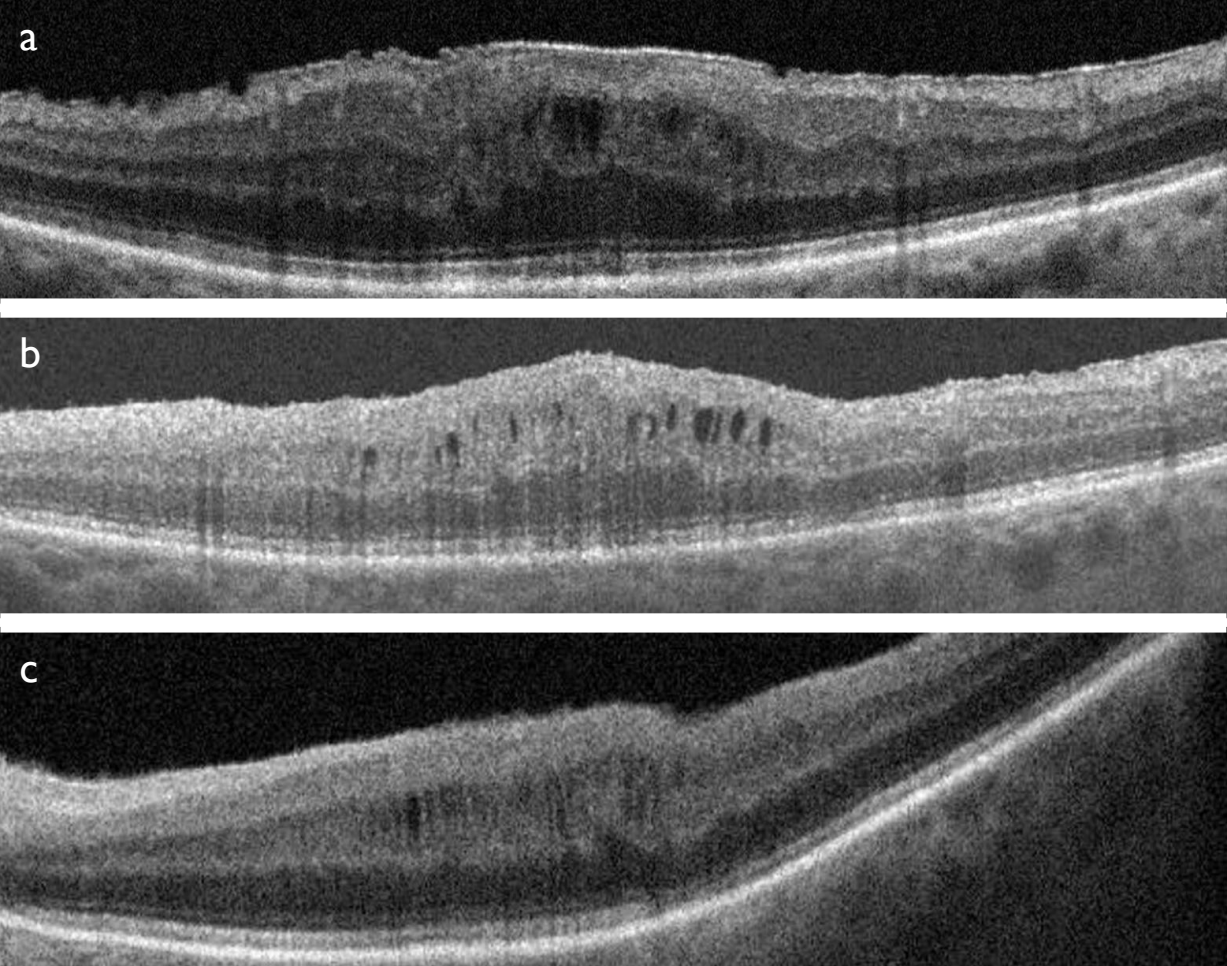
**(a)** Preoperative optical coherence tomography of epiretinal membrane. **(b)** Early postoperative optical coherence tomography after epiretinal membrane peeling. **(c)** Late postoperative optical coherence tomography after epiretinal membrane peeling.

In five eyes with ERM, the ERM and ILM were removed successfully (Fig. 1). In one eye which was operated for MH, the hole closure was achieved at the last visit.

Post-operative complications in different surgical indications are summarized in Table 3.

**Table 3.** Postoperative complications

**Complications**	**n (%)**	**According to Surgical Indication**	**Intervention**
Early complications (within 1 month)			
Early postoperative VH	9 (21.4)	3 VH	Spontaneous resolution
		2 TRD	
		1 SMH	
		3 TRD+VH	
Transient severe hypotony (<5 mmHg)	1 (2.3)	1 VH	Spontaneous resolution
Retinal detachment	2 (4.7)	2 TRD	Re-vitrectomy
Late complications (after 1 month)			
Late postoperative VH	2 (4.7)	1 VH	Re-vitrectomy
		1 TRD+VH	
Recurrent retinal detachment	3 (7.1)	3 TRD+VH	1 Observation
			2 Re-vitrectomy

N: number of eyes; VH: vitreous hemorrhage; TRD: tractional retinal detachment; SMH: submacular hemorrhage.

## Discussion

23G TSV technique is one of the most important steps in vitreoretinal surgery, which enables sutureless PPV by not requiring conjunctival peritomy or closing sclerotomies. The advantage of the sutureless vitrectomy system is based on the reduction of trauma caused by surgery in sclerotomy sites, enabling self-closing sclerotomies, and accelerating post-operative recovery. Although 23G PPV technique offers sutureless transconjunctival surgery similar to 25G PPV system, it is similar to the classical 20G PPV system allowing the usage of less flexible instruments. In this study, the results of 23G TSV in patients with VH, TRD, TRD+VH, ERM, DME, SMH, MH, and VMT due to DR were examined. ERM and ILM peeling, bimanual membrane dissection, endolaser photocoagulation, and endotamponade injection were successfully performed in all cases.

Sutures which are used to close the sclerotomies cause irritation and pigmentation in the sclerotomy sides (8). In 23G TSV surgery, sclerotomy incision is performed in a way to form a bevel, self-closing tunnel, which prevents suture-related irritation and local inflammatory reaction in sclerotomy sites. In this study, only one sclerotomy site in each of eight eyes and three sites in each of three eyes had to be sutured due to leakage.

There was a significant increase in post-operative visual acuity at the 1^st^ month, 3^rd^ month, and final visit in the whole study group. In subgroup analysis, a significant improvement in vision was achieved for eyes with DME and non-clearing VH. There was no significant increase in vision in the eyes which were operated for TRD. Studies have reported that residual subfoveal fluid after a successful surgery affects functional recovery in patients with TRD (9). In our study, serous macular detachment developed in two eyes and retinal detachment in two eyes. The cause of recurrent retinal detachment was unreleased retinal tractions. Because of these cases, a significant functional improvement could not be obtained in patients with TRD. Revitrectomy was performed in eyes with retinal detachment, but observation without intervention was preferred in eyes with serous macular detachment.

Combined phacoemulsification with 23G TSV was applied to seven eyes without any complication. It has been reported in the literature that combined phacoemulsification with 23G TSV is an effective and safe method in vitreoretinal diseases. Likewise, sutureless vitrectomy results in faster vision enhancement and less ocular inflammation (10,11).

Hypotonia, even if it is temporary, is a dangerous condition in terms of complications that may develop postoperatively. It increases the probability of suprachoroidal hemorrhage and endophthalmitis. The previous studies reported that transient post-operative hypotony defined as IOP of less than 10 mmHg ranged from 21.1% to 26.6% in 23G TSV (12,13). In the present study, transient post-operative hypotony occurred in 6 (14.28%) eyes in the early post-operative period, but there was no evidence of choroidal detachment or related complications and no treatment was necessary. IOP was above 10 mmHg at the end of the 1^st^ week in all cases. In these cases, wounds were found to be intact in the slit-lamp examination. No cases of endophthalmitis have been observed. This may be due to the superior wound structure with a longer and beveled scleral tunnel produced with the 23G system. Furthermore, the wound architecture created with an angled incision is less likely to provide a way for the bacteria to enter the eye. The previous studies have reported the incidence of early post-operative VH between 0% and 1.2% in 23G TSV (12,14,15). In this study, VH developed in nine eyes in the early post-operative period. The reason for the high rate of early VH compared to other studies was probably due to the number of diabetic patients which was between 0% and 19.7% in the previous studies, whereas all patients had DR in the current study. In all of these nine eyes, VH resorbed spontaneously within 1 month. These were considered to be the hemorrhages from the sclerotomy sites (16).

In the late post-operative period, revitrectomy with 23G TSV was performed in two eyes with recurrent VH and in two eyes with recurrent retinal detachment. In one eye with recurrent retinal detachment, the preferred intervention was observation. The cause of recurrent retinal detachment in these cases was fibrous proliferation. The frequency of retinal detachment was found to be 2.2% in the series of Ibarra et al., using 25G PPV, (17) and 2% in the series of Fujii et al.(6) In the 23G PPV series of Fine et al., no tear and retinal detachment were detected in any patient.[18] In the series of this study, revitrectomy was performed for recurrent retinal detachment in four eyes, two in the early post-operative period and two in the late period. Retina was attached and flat in all eyes at the follow-up time.

Limitations of this study are its retrospective nature and the inclusion of uncontrolled, non-comparative case series with a small sample size.

## CONCLUSION

This study demonstrates that 23G TSV surgery is an effective technique for the treatment of vitreoretinal conditions due to complications of DR in selected cases and the desired anatomical and functional success can be achieved.

## Disclosures

### Ethics Committee Approval:

Prof.Dr. Cemil Tascioglu City Hospital, E-48670771-514.10.

### Peer-review:

Externally peer-reviewed.

### Conflict of Interest:

None declared.

### Authorship Contributions:

Involved in design and conduct of the study (IP); preparation and review of the study (IP); data collection (IP, MC); and statistical analysis (IP, MC).
